# Partnering with a senior living community to optimise teledermatology via full body skin screening during the COVID‐19 pandemic: A pilot programme

**DOI:** 10.1002/ski2.141

**Published:** 2022-06-27

**Authors:** Pavin Trinh, Kiana Yekrang, Michelle Phung, Silvina Pugliese, Anne Lynn S. Chang, Elizabeth E. Bailey, Justin M. Ko, Kavita Y. Sarin

**Affiliations:** ^1^ Department of Dermatology Stanford University School of Medicine Stanford California USA

## Abstract

**Background:**

Elderly patients in senior communities faced high barriers to care during the COVID‐19 pandemic, including increased vulnerability to COVID‐19, long quarantines for clinic visits, and difficulties with telemedicine adoption.

**Objective:**

To pilot a new model of dermatologic care to overcome barriers for senior living communities during the COVID‐19 pandemic and assess patient satisfaction.

**Methods:**

From 16 November 2020 to 9 July 2021, this quality improvement programme combined in‐residence full body imaging with real‐time outlier lesion identification and virtual teledermatology. Residents from the Sequoias Portola Valley Senior Living Retirement Community (Portola Valley, California) voluntarily enroled in the Stanford Skin Scan Programme. Non‐physician clinical staff with a recent negative COVID‐19 test travelled on‐site to obtain in‐residence full body photographs using a mobile app‐based system on an iPad called SkinIO that leverages deep learning to analyse patient images and suggest suspicious, outlier lesions for dermoscopic photos. A single dermatologist reviewed photographs with the patient and provided recommendations via a video visit. Objective measures included follow‐up course and number of skin cancers detected. Subjective findings were obtained through patient experience surveys.

**Results:**

Twenty‐seven individuals participated, three skin cancers were identified, with 11 individuals scheduled for a follow up in‐person visit and four individuals starting home treatment. Overall, 88% of patients were satisfied with the Skin Scan programme, with 77% likely to recommend the programme to others. 92% of patients agreed that the Skin Scan photographs were representative of their skin. In the context of the COVID‐19 pandemic, 100% of patients felt the process was safer or comparable to an in‐person visit. Despite overall appreciation for the programme, 31% of patients reported that they would prefer to see dermatologist in‐person after the pandemic.

**Conclusions:**

This programme offers a framework for how a hybrid skin scan programme may provide high utility for individuals with barriers to accessing in‐person clinics.

## Graphical abstract

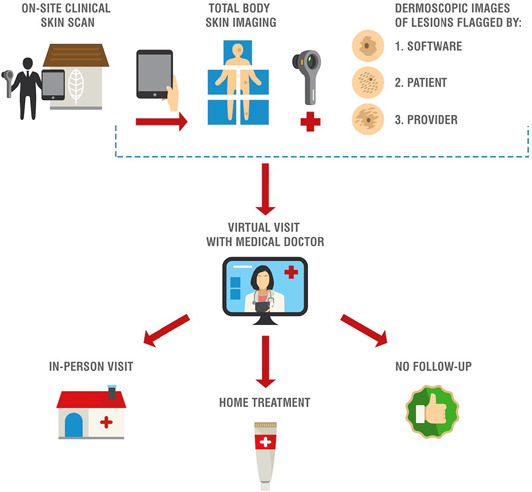

1

**What is already known about this topic?**

- Elderly patients are at high risk of developing skin cancers and rely on skin screening exams for early skin cancer detection and treatment.
- During the COVID‐19 pandemic, difficulty with adopting traditional teledermatology methods, quarantines for clinic visits, and high vulnerability to COVID‐19 disproportionately increased barriers to care for elderly populations, especially those in senior living communities.

**What does this study add?**

- Traditional methods of teledermatology are unable to overcome these new barriers to care in the context of a pandemic and the vulnerable population in senior living communities.
- The Stanford Skin Scan Programme advances traditional teledermatology models by combining store‐and‐forward and live‐interactive models, with high quality images captured through a neural network‐based application and dermatologists equipped with images to synchronously screen patients over video.

**What are the clinical implications of this work?**

- We offer a method of increasing access to skin cancer screening while ensuring COVID‐19 safety, bringing direct‐access care to patients lacking mobility, and optimising skin screening by capturing high quality photographs.
- The Stanford Skin Scan Programme serves as a framework for a hybrid approach to combine remote care capabilities to meet the unique needs of vulnerable patients, especially those facing barriers to dermatologic care during a pandemic.

## 2 INTRODUCTION

Starting in March 2020, the COVID‐19 pandemic disproportionately affected the health and safety of elderly populations across the United States. Individuals over 65 years old made up 76%–81% of COVID‐19 deaths.^1^ Senior living facilities were most significantly impacted, constituting 40% of COVID‐19 deaths in the United States.^2^ Strict protocols to reduce potential COVID‐19 exposure created health care access barriers for seniors with a 56%–85% decrease in preventative cancer screens.^3^ This similar effect was seen across dermatology clinics. The number of elderly patients who visited Stanford Dermatology clinics declined by 37% during the pandemic compared to the previous year.^4^ There was also a 23% decline in the number of skin cancers diagnosed among the elderly.^4^ As elderly patients are at highest risk of developing skin cancer, with 74% of new melanomas found in those over the age of 55,^5^ ^,^ ^6^ we sought to develop a pilot programme to circumvent the obstacles to health care and prevent delays in cancer diagnosis for elderly living in senior living facilities.

In the current landscape, a patient can see a physician either through (1) an in‐person visit, (2) a house call, or (3) a telemedicine visit. For each of these options, we assessed barriers to care affecting our independent seniors in retirement communities. In‐person visits allow for direct care, but are restrictive for patients who have mobility issues, lack reliable means of transportation, have post‐visit quarantine requirements, or fear risking exposure to COVID‐19.^7^, ^8^, ^9^ Specialist house calls address patient transportation issues, but can be clinically inefficient and costly due to the extra time required for travel to and between patients, reducing the total number of patients that a dermatologist can serve, which is especially detrimental in the United States where average dermatology wait times can be as high as 50 days.^10^, ^11^, ^12^ Teledermatology addresses the logistical constraints of the previous methods, but can be significantly limited by poor quality photographs.^13^ Effective teledermatology screening requires high‐quality full body and close‐up photographs for accurate evaluation.^13^ In traditional teledermatology, these photographs are difficult to obtain because patients, such as the elderly, may lack the required digital literacy, access to technology, or ability to capture photos of difficult to reach areas if they live alone.^14^, ^15^ Patients with limited dexterity, tremors, cognitive impairment, or visual impairment may face challenges with operating technology and capturing high‐quality photographs required for teledermatology triage.^14^ While the COVID‐19 pandemic led to rapid adoption of telemedicine in the general population, elderly patients accounted for only 8% of total teledermatology visits at Stanford dermatology clinics during the pandemic.^4^ Previous research has shown that overcoming barriers to teledermatology due to need for high quality photos and/or dermoscopy in the elderly population can be accomplished by technical assistance and electronic equipment to image the skin in 66% of patients.^16^

To address the major limitations of traditional care models, we designed a programme that would ensure COVID‐19 safety, bring direct‐access care to patients lacking mobility, and improve upon current teledermatology methods by capturing high quality photographs to optimise skin screening. From 16 November 2020 to 9 July 2021, we piloted a novel method of delivering dermatologic care to a local senior living community during the COVID‐19 pandemic using guided, in‐home skin scanning and outlier lesion identification. Through clinical data and patient experience surveys, we sought to understand how a hybrid skin scan programme incorporating high‐quality remote photography with dermoscopy might offer value to vulnerable patients with difficulty accessing dermatologic care. In addition to designing a novel workflow to address barriers to care, our objective was to assess patient satisfaction with this programme.

## 3 PATIENTS AND METHODS

### 3.1 Study population

Between 16 November 2020 to 9 July 2021, residents at the Sequoias Portola Valley Senior Living Retirement Community were invited to participate in the Stanford Skin Scan Programme. Information about the programme was distributed through flyers and word of mouth at the senior living community. Residents interested in participating contacted research staff to schedule an appointment for an on‐site full body skin scan and a subsequent video visit with a dermatologist. This programme was exempt from the Stanford University Institutional Review Board as an observational standard of care project. This manuscript followed the Standards for Reporting Qualitative Research (SRQR) recommendations.

### 3.2 Study design and data sources

**FIGURE 1:**
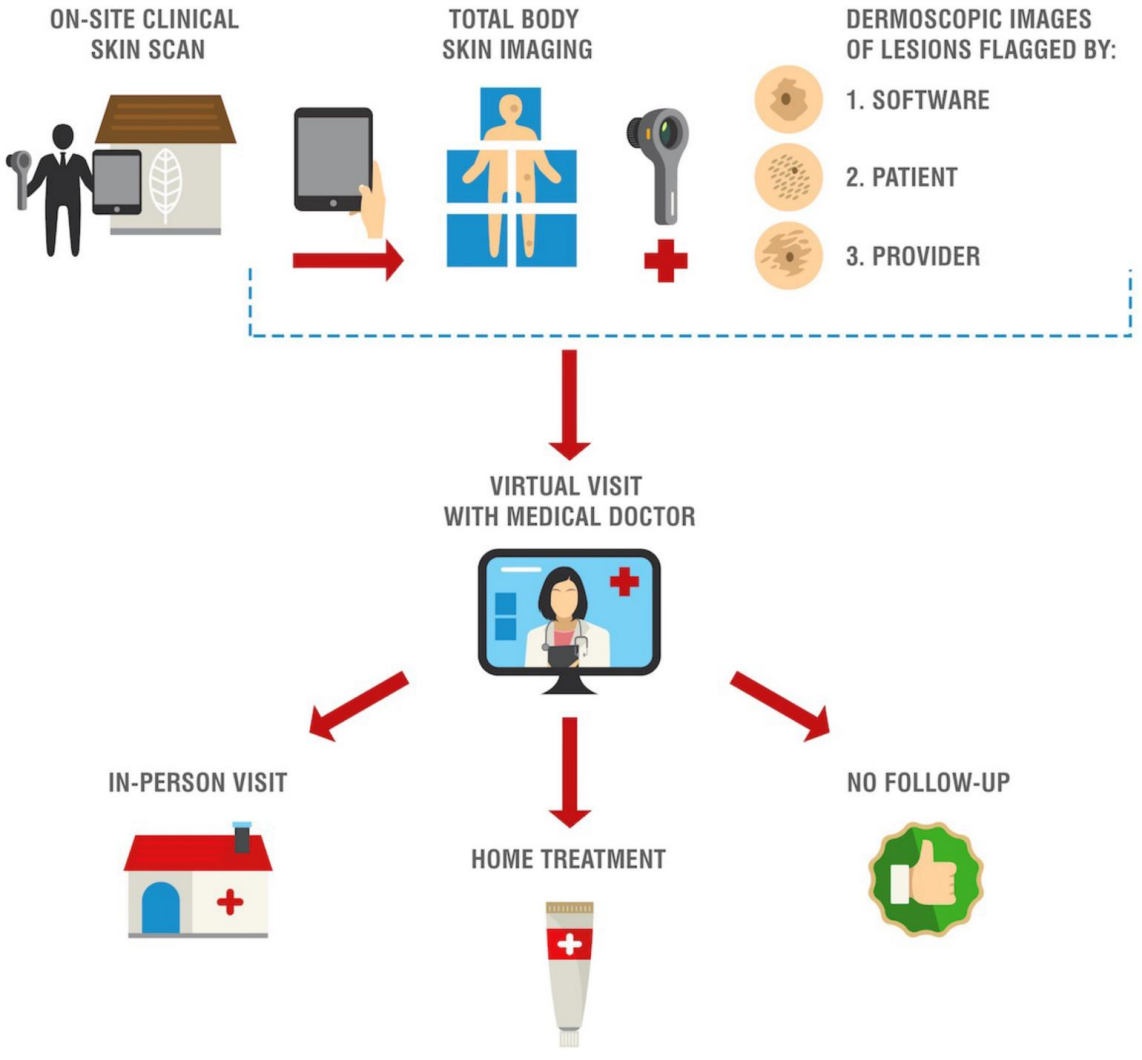
Stanford skin scan programme

**FIGURE 2:**
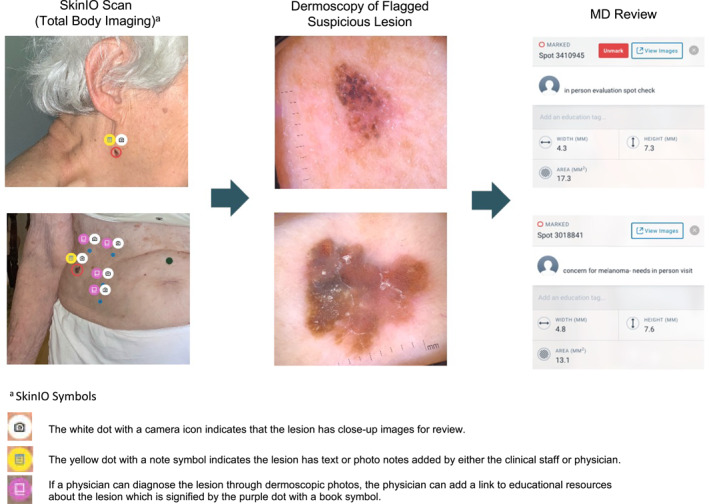
SkinIO symbols

From 16 November 2020 to 9 July 2021, a total of 27 residents participated in the Stanford Skin Scan Programme. Non‐physician clinical staff travelled on‐site to senior residences to obtain full body photographs (Figure 1). In accordance with the Sequoias Portola Valley's COVID‐19 regulations, clinical staff were required to provide proof of a negative COVID‐19 test 24–72 h prior to visitation, complete a health survey, and wear personal protective equipment when entering residents' homes. Clinical staff wore gowns, KN95 masks, face shields, and gloves while obtaining images. These non‐physician clinical staff were trained on how to use the iPad Pro, dermatoscope, and SkinIO application to obtain full body photographs. SkinIO was used as a tool for image collection, with a streamlined interface enabling the physician to quickly browse through organised collections of full body and dermoscopic photos (Figure 2).^17^, ^18^ The application uses a deep neural network‐based outlier detection model that analyzes all photos from a patient's total body photography session, compares each lesion, and highlights lesions most different from the average. The neural network aspect was only used in triage for photography, but not for diagnosis. All diagnoses were made by the physician upon clinical review of photographs. Upon review of the full body and dermoscopic photos, a physician could still request additional dermoscopic photos to be taken at a future visit. Lesions were either flagged by the SkinIO software in real‐time, by the patient in real‐time, or by the treating physician upon final review of all photos. Dermoscopic images were obtained for all flagged lesions with a DermLite DL1 attachment. If the lesion was flagged by the treating physician, clinical staff would obtain additional dermoscopic images the next time they visited the retirement community. Afterwards, patients were scheduled for a virtual visit with a Stanford dermatologist. During the virtual visit, a dermatologist reviewed photographs with patients, addressed patient questions or concerns, and provided recommendations. These recommendations could either be an in‐person visit for further workup, home treatment, or reassurance that follow‐up was not necessary. Participants were also given USB hard drives, printouts of their photos, and providers' notes on specific lesions for their records. In total, this pilot study required 2 clinical staff and two iPad Pros, each with a DermLite DL1 attachment and the SkinIO application downloaded.

Upon completion of the full body scan and video visit, each patient was asked to complete a 16‐question survey about their experience with the Stanford Skin Scan Programme (Supplemental 1). The participating provider also completed a 16‐question survey about their experience (Supplemental 2).

### 3.3 Statistical analysis

Descriptive statistics summarising means and standard deviations of patient demographics as well as frequencies of survey responses were calculated. Survey results were calculated based on answered questions. Data analysis was completed using Microsoft Excel, version 16.53 (Microsoft).

## 4 RESULTS

Of the 27 participants in the Stanford Skin Scan Programme, 21 were female (77%). The mean (SD) age was 87 (5.4) years (range, 79–99 years). 26 were white (96%). The median household income of the area was $220 000.

### 4.1 Clinical outcomes

**FIGURE 3:**
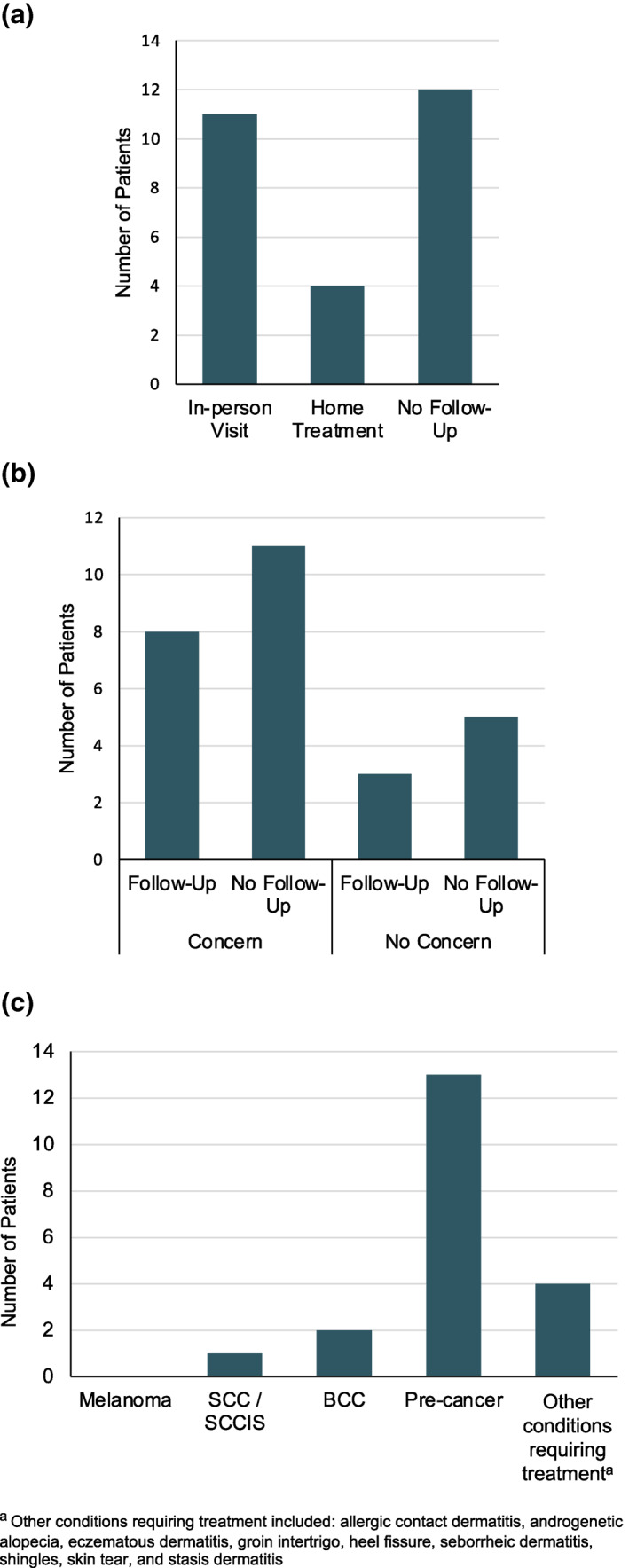
(a) Next step after skin scan and video visit; (b) Frequency of in‐person follow‐up by patients with and without concerns; (c) Distribution of skin lesions examined

Of the 27 patients triaged, most patients (16 [59%]) were successfully managed without requiring an in‐person follow‐up visit (Figure 3). All patients who were required to have further examination in person were seen in person, resulting in a 0% attrition rate. Of the 19 patients with a specific skin concern prior to their telehealth visit, 16 patients had a skin issue requiring treatment. Of these 19 patients with concerns, eight were asked to follow‐up in person and 11 did not require follow‐up. Of the eight patients who did not have a specific skin concern, three were asked to follow‐up in person and five did not require follow‐up. Patients requiring further examination in person or procedures were asked to follow‐up in person.

Of the dermoscopically imaged lesions, 63% (186) were flagged by the SkinIO application and 33% (97) were flagged by the patient. The remaining 4% (12) were flagged by the physician. While the physician did not label each of the 186 dermoscopic images due to time, the majority were of benign lesions. Of these 186 lesions, 13 were actinic keratoses, two were basal cell carcinomas, and one was a squamous cell carcinoma. Other encountered conditions requiring treatment included allergic contact dermatitis, androgenetic alopecia, eczematous dermatitis, groin intertrigo, heel fissure, hyperkeratosis of heels, seborrhoeic dermatitis, shingles, skin tears, and stasis dermatitis.

### 4.2 Survey outcomes

Participants were surveyed about their experience with the Stanford Skin Scan Programme by phone or paper survey after completing their video visit with the dermatologist (Figure 4). 26 out of 27 patients completed the survey for a 96% response rate. 83% of participants felt the comprehensiveness or quality of the programme was comparable or better to an in‐person dermatology visit. 81% of participants felt that their ability to connect with the dermatologist and ask questions was comparable to or better than an in‐person visit. In the context of the COVID‐19 pandemic, participants felt that the full body skin imaging (100%) and the video visit (96%) were safer or comparable to an in‐person visit. In the absence of the programme, 38% of participants would not have sought care.

FIGURE 4(a) Stanford Skin Scan Programme compared to in‐person visits; (b) Overall Stanford Skin Scan Programme assessments; (c) Under what circumstances would you use the Skin Scan Programme after the COVID‐19 pandemic? (d) What would you have done if you had not been able to participate in the Skin Scan Programme?

**Figure:**
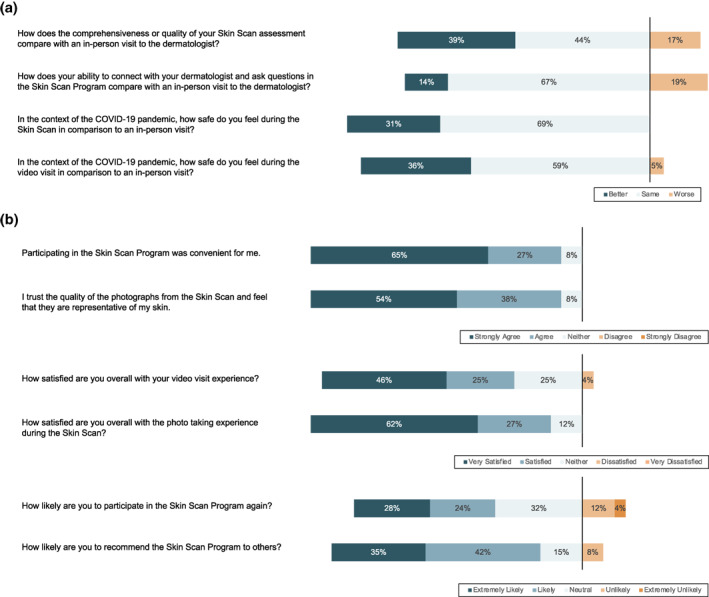

**Figure:**
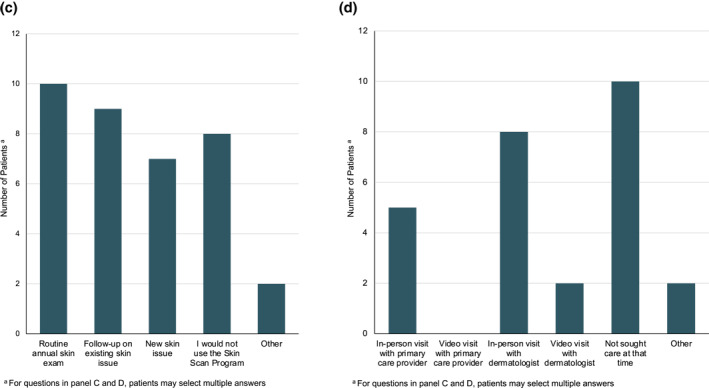

Ninety‐two percent of participants either strongly agreed or agreed that the Stanford Skin Scan Programme was convenient, trustworthy, and representative of their skin. Participants were satisfied with the full body imaging (88%) and the video visit experience (71%). 77% of participants were extremely likely or likely to recommend the programme to others and 52% were extremely likely or likely to participate in the Stanford Skin Scan Programme again. 30% of participants reported that they would prefer to see an in‐person dermatologist instead of the Stanford Skin Scan programme after the pandemic. Of those who planned to use the Stanford Skin Scan programme after the COVID‐19 pandemic was resolved, 38% intended use for routine annual exam, 35% for follow‐up on existing skin issues, and 27% for new skin issues.

Based on the physician survey (*n* = 1), the treating dermatologist was overall very satisfied, trusted the quality of her skin assessment, and felt that providing care through the programme was safer than an in‐person visit. She felt that the quality and comprehensiveness of the programme's photographs were superior to patient provided photographs in traditional video visits. While she was extremely likely to offer patients the Stanford Skin Scan programme during the COVID‐19 pandemic, the physician was neutral about offering this programme to all patients after the pandemic, citing that an in‐person visit felt more reliable and that the programme may be a good alternative for patient's lacking clinic access.

## 5 DISCUSSION

This pilot programme illustrates a novel model for delivering dermatologic care to a senior living community during the COVID‐19 pandemic. Our programme addresses several barriers to care seen in traditional care models for this vulnerable population. To bring direct‐access care to patients lacking mobility or access to transportation, non‐physician clinical staff travelled directly on‐site and all patients saw a dermatologist virtually. To ensure COVID‐19 safety, clinical staff were required to have a negative COVID‐19 test and wear personal protective equipment. To obtain high quality full body and dermoscopic photos, we leveraged SkinIO's easy‐to‐use interface for image collection and organization, removing the onus on the patient to capture high quality photos. The mobile application utilised a deep neural network‐based outlier identification system which flagged suspicious lesions, enabling clinical staff to take focussed high‐resolution photographs in real‐time without physician guidance. SkinIO was used as a tool for image collection and to assist non‐physician staff in triaging which lesions to take dermoscopic photos of. In all scenarios, final diagnoses and clinical decision making, including if additional lesions should be dermoscopically imaged, were made by a dermatologist and not SkinIO. Using high quality full body and dermoscopic photographs, we were able to recapitulate an in‐person exam and enable skin screening via video. With regards to our primary objective of assessing patient satisfaction with this workflow, patients felt the Stanford Skin Scan Programme was representative of their skin and safer or comparable to an in‐person visit. Overall, patients agreed that the programme was beneficial and would recommend it to others, especially individuals with mobility issues.

Teledermatology has developed into two primary forms: store‐and‐forward and live‐interactive.^19^ Store‐and‐forward teledermatology involves medical support staff or referring primary care physicians capturing and sending images for a remote dermatologist to asynchronously review without the patient present.^20^ Live‐interactive teledermatology involves a synchronous visit between patient and dermatologist over video.^20^ A major drawback of a store‐and‐ forward model is the lack of interaction with a specialist.^21^ The Stanford Skin Scan Programme advances these traditional models by combining elements of both store‐and‐forward and live‐interactive, with high quality images captured through a neural network‐based application and dermatologists equipped with images to synchronously screen patients over video.

Patient satisfaction with the Stanford Skin Scan Programme was consistent with studies looking at both store‐and‐forward and live‐interactive models, which excel in offering flexibility for the patient and confidence in clinical care.^22^, ^23^ In the context of the COVID‐19 pandemic, patients report choosing teledermatology for its efficiency, accessibility without transportation, and ability to maintain social distancing.^24^ Despite these strengths, 31% of patients in our programme would rather see a dermatologist in‐person after the pandemic, aligning with rates of 30%–42% from other studies.^25^, ^26^ Patient preference for in‐person visits may be attributed to perceived thoroughness of physically feeling lesions and ability to obtain on‐site intervention as well as the desire for in‐person interactions with their physician.^24^

Despite current methods of teledermatology, the COVID‐19 pandemic created material delays in skin cancer diagnoses, predisposing patients to develop more advanced disease.^27^, ^28^ Teledermatology that directly addresses barriers to care for elderly patients may promote earlier skin cancer diagnosis and treatment in those who would have otherwise not sought care.^29^ Due to the severe shortage of dermatologists in the United States for the past several decades,^30^ the ability to conduct visits for patients with mobility issues without requiring dermatologists onsite is critical to providing care for this population. By training non‐physician clinical staff to travel on‐site and obtain high quality photographs, our pilot programme enables patients with mobility issues to access specialist care in the context of the severe dermatologist shortage and need for high quality photographs for appropriate triage. Of the participants in the programme, 38% would not have sought care if the programme were not available. On average, patients who required an in‐person visit were seen in 24 days through the programme compared to 58 days through standard booking for in‐person appointments. Along with earlier diagnosis, implementing teledermatology for triage has been shown to result in earlier clinical resolution by up to 26 days^31^ Furthermore, there is a role for teledermatology to reduce unnecessary in‐person visits and COVID‐19 exposure.^32^ Our programme reassured 58% of patients with a skin concern through virtual visit, saving them an unnecessary in‐person visit, a prolonged quarantine, and needless COVID‐19 exposure. Similarly, a store‐and‐forward teledermatology model in Philadelphia found a reduction of in‐person dermatology visits by 27%.^33^

Of note, the Skin Scan Programme did require more provider time than a traditional 15 min in‐person visit. About 35 min of clinical staff time was required for full body photography and dermoscopy. A total of 20 min of provider time was required, with 5 min to review photographs and 15 min of virtual facetime. There were also upfront direct costs such as time spent training clinical staff to capture high quality photos and the purchase of an iPad and a dermatoscope.

While the additional time and resources spent may not be suitable for the general dermatologic population, the Stanford Skin Scan Programme may provide greatest utility for certain vulnerable populations, such as skilled nursing facilities, deployed military personnel, and those with mobility issues. From a systems perspective, the additional direct costs required to provide care for vulnerable populations should be weighed against the broader societal costs of solely providing in‐person care.^34^ Some societal costs include travel time, loss of productivity or leisure time, and the consequences of delayed diagnoses for patients unable to attend in‐person visits.^34^ Randomized controlled trials of both store‐and‐forward and live‐interactive teledermatology have found that, when societal costs are factored, teledermatology was overall cost‐effective with per‐patient savings of $82 and $145, respectively.^35^, ^36^ In elderly and frail populations, societal costs should include the costs of caretakers who accompany the patient, specialised transportation for those with mobility issues, and severe downstream complications of contracting COVID‐19.^31^, ^37^ Offering teledermatology to the most vulnerable tier of patients may enable the greatest cost savings, given the greater societal costs of in‐person care. Future research should evaluate the cost effectiveness of incorporating mobile applications, such as SkinIO, in these modes of care and in different populations of need.

There were several limitations inherent to a single site study. Our mainly white, higher income, English‐speaking, and more educated patient cohort is less generalisable to the broader elderly population who may deal with additional financial or language barriers. The programme size was limited by the small number of patients and a single provider who volunteered to participate which reduces the generalisability. Future studies would benefit by including more participants across multiple, diverse senior living communities and additional providers. Additionally, patients in other studies have objected to skin photography due to social, religious, or privacy concerns.^25^, ^38^ This viewpoint may be underrepresented in our survey results which represent a volunteer sample comfortable with being photographed. With regards to full body skin imaging, only certain areas could be captured by photography. Areas such as the scalp and groin were more difficult to accurately capture with appropriate lighting. A trained photographer was needed to capture the images on site.

It is important to acknowledge that the SkinIO application was not intended or used for clinical diagnosis. As such, our programme objective was not to assess diagnostic efficacy of the application. Guido et al. previously showed that SkinIO had 92% sensitivity in detecting new or changing lesions.^17^ Future studies can explore the role mobile applications may play in remote diagnosis.

To our knowledge, this is the first implementation of a full body skin scan programme for a senior living community during the COVID‐19 pandemic, with high rates of satisfaction. The Stanford Skin Scan Programme is a model framework for a hybrid approach leveraging remote care capabilities to meet the unique needs of vulnerable patients and maintain the critical continuum of dermatologic care for patients at highest risk of skin cancer.

## AUTHOR CONTRIBUTIONS

**Pavin Trinh:** Formal analysis (Lead); Investigation (Lead); Methodology (Equal); Validation (Equal); Visualization (Equal); Writing – original draft (Lead); Writing – review & editing (Lead). **Kiana Yekrang:** Conceptualization (Equal); Data curation (Lead); Investigation (Lead); Methodology (Lead); Project administration (Lead); Resources (Equal); Software (Equal); Validation (Equal); Visualization (Equal); Writing – original draft (Supporting); Writing – review & editing (Supporting). **Michelle Phung:** Conceptualisation (Equal); Data curation (Equal); Investigation (Equal); Project administration (Equal); Resources (Equal); Software (Equal). **Silvina Pulgies:** Conceptualization (Supporting); Data curation (Supporting); Formal analysis (Supporting); Funding acquisition (Supporting); Investigation (Supporting); Methodology (Supporting); Project administration (Supporting); Visualisation (Supporting); Writing – review & editing (Supporting). **Anne Lynn Chang:** Conceptualization (Supporting); Data curation (Supporting); Formal analysis (Supporting); Funding acquisition (Supporting); Investigation (Supporting); Methodology (Supporting); Project administration (Supporting); Visualization (Supporting); Writing – review & editing (Supporting). **Elizabeth Bailey:** Conceptualization (Supporting); Data curation (Supporting); Formal analysis (Supporting); Funding acquisition (Supporting); Investigation (Supporting); Methodology (Supporting); Project administration (Supporting); Visualization (Supporting); Writing – review & editing (Supporting). **Justin M. Ko:** Conceptualisation (Equal); Data curation (Equal); Formal analysis (Equal); Funding acquisition (Equal); Investigation (Equal); Methodology (Equal); Project administration (Equal); Resources (Equal); Software (Equal); Supervision (Equal); Validation (Equal); Visualization (Equal); Writing – review & editing (Equal). **Kavita Yang Sarin:** Conceptualisation (Equal); Data curation (Equal); Formal analysis (Equal); Funding acquisition (Equal); Investigation (Equal); Methodology (Equal); Project administration (Equal); Resources (Equal); Software (Equal); Supervision (Equal); Validation (Equal); Visualization (Equal); Writing – original draft (Equal); Writing – review & editing (Equal).

## CONFLICT OF INTEREST

The author declares that there is no conflict of interest that could be perceived as prejudicing the impartiality of the research reported.

## ETHICS STATEMENT

This programme was exempt from the Stanford University Institutional Review Board as an observational standard of care project. This manuscript followed the Standards for Reporting Qualitative Research (SRQR) recommendations.

## Supporting information

Supplementary Material

Supplementary Material

## Data Availability

The data that support the findings of this study are available from the corresponding author upon reasonable request.
