# Supplementary material for: Partnering with a senior living community to optimise teledermatology via full body skin screening during the COVID‐19 pandemic: A pilot programme
Source: Skin Health Dis. 2022 Jun 27;2(3):e141. doi: 10.1002/ski2.141 (PMC9349994; doi:10.1002/ski2.141)
Supplement: Supplementary file 1 — Supplementary Material [file SKI2-2-e141-s001.pdf]

Supplemental 1. Patient Survey

Skin Scan Survey

1. I trust the quality of the photographs from the Skin Scan and feel that they are representative of my skin.
   - ☐ Strongly agree
   - ☐ Agree
   - ☐ Neither agree nor disagree
   - ☐ Disagree
   - ☐ Strongly disagree

2. How does the comprehensiveness or quality of your Skin Scan assessment compare with an in-person visit to the dermatologist?
   - ☐ Better
   - ☐ Same
   - ☐ Worse
   - ☐ Not sure

3. How does your ability to connect with your dermatologist and ask questions in the Skin Scan Program compare with an in-person visit to the dermatologist?
   - ☐ Better
   - ☐ Same
   - ☐ Worse
   - ☐ Not sure

4. In the context of the COVID-19 pandemic, how safe do you feel during the Skin Scan in comparison to an in-person visit?
   - ☐ Better
   - ☐ Same
   - ☐ Worse
   - ☐ Not sure

5. Participating in the Skin Scan Program was convenient for me.
   - ☐ Strongly agree
   - ☐ Agree
   - ☐ Neither agree nor disagree
   - ☐ Disagree
   - ☐ Strongly disagree

6. How satisfied are you overall with the photo taking experience during the Skin Scan?
   - ☐ Very satisfied
   - ☐ Satisfied

- ☐ Neither satisfied nor dissatisfied
- ☐ Dissatisfied
- ☐ Very dissatisfied

7. Please state any additional feedback on your experience with the Skin Scan or other ways we can improve:

____
____
____
____
____
____

Video Visit Survey

8. Under what circumstances would you use the Skin Scan Program during the COVID-19 pandemic?
   - ☐ Routine annual skin exam
   - ☐ Follow-up on an existing skin issue
   - ☐ New skin issue
   - ☐ Other (please specify):____

9. Under what circumstances would you use the Skin Scan Program after the COVID-19 pandemic?
   - ☐ Routine annual skin exam
   - ☐ Follow-up on an existing skin issue
   - ☐ New skin issue
   - ☐ I would not use the Skin Scan Program
   - ☐ Other (please specify):____

10. What would you have done if you had not been able to participate in the Skin Scan Program?
    - ☐ Book an in-person visit with my Primary Care Provider
    - ☐ Book a video visit with my Primary Care Provider
    - ☐ Book an in-person visit with my Dermatologist
    - ☐ Book a video visit with my Dermatologist
    - ☐ I would not have sought care at that time
    - ☐ Other (please specify):____

11. In the context of the COVID-19 pandemic, how safe do you feel during the video visit in comparison to an in-person visit?
    a. Better
    b. Same

c. Worse
d. Not sure

12. How likely are you to participate in the Skin Scan Program again?
    - ☐ Extremely likely
    - ☐ Likely
    - ☐ Neutral
    - ☐ Unlikely
    - ☐ Extremely unlikely

13. How likely are you to recommend the Skin Scan Program to others?
    - ☐ Extremely likely
    - ☐ Likely
    - ☐ Neutral
    - ☐ Unlikely
    - ☐ Extremely unlikely

14. How satisfied are you overall with your video visit experience?
    - ☐ Very satisfied
    - ☐ Satisfied
    - ☐ Neither satisfied nor dissatisfied
    - ☐ Dissatisfied
    - ☐ Very dissatisfied

15. Please state any additional feedback on your experience with the video visit or other ways we can improve:

____
____
____
____
____

16. Do you give Stanford permission to follow-up with you on any suggestions you provided for further information?
    - ☐ Yes

      Please provide your name and the best phone number to reach you at:
      ____
    - ☐ No
