# Supplementary material for: Partnering with a senior living community to optimise teledermatology via full body skin screening during the COVID‐19 pandemic: A pilot programme
Source: Skin Health Dis. 2022 Jun 27;2(3):e141. doi: 10.1002/ski2.141 (PMC9349994; doi:10.1002/ski2.141)
Supplement: Supplementary file 2 — Supplementary Material [file SKI2-2-e141-s002.pdf]

Supplemental 2. Provider Survey

1. I trust the quality of the skin assessments that I performed with the Skin Scan Program.
   - ☐ Strongly agree
   - ☐ Agree
   - ☐ Neither agree nor disagree
   - ☐ Disagree
   - ☐ Strongly disagree

2. How does the comprehensiveness or quality of an in-person skin assessment compare with a video visit assessment?
   - ☐ Better
   - ☐ Same
   - ☐ Worse
   - ☐ Not sure

3. How does the comprehensiveness of an in-person skin assessment compare with a video visit assessment that includes Skin Scan photographs?
   - ☐ Better
   - ☐ Same
   - ☐ Worse
   - ☐ Not sure

4. How does the comprehensiveness of a video visit assessment (on its own) compare with a video visit assessment that includes Skin Scan photographs?
   - ☐ Better
   - ☐ Same
   - ☐ Worse
   - ☐ Not sure

5. In the context of the COVID-19 pandemic, as a provider, how safe do you feel during the Skin Scan assessments in comparison to an in-person visit?
   - ☐ Better
   - ☐ Same
   - ☐ Worse
   - ☐ Not sure

6. In the context of the COVID-19 pandemic, as a provider, how do you think the safety of patients during the Skin Scan assessments compares to an in-person visit?
   - ☐ Better
   - ☐ Same
   - ☐ Worse

- ☐ Not sure

7. How much time, on average, does it take you to complete an in-person assessment of a patient? ____ minutes.

8. How much time, on average, does it take you to complete a video assessment of a patient? ____ minutes.

9. How much time, on average, does it take you to complete a video assessment, including photographs from the Skin Scan, of a patient? ____minutes.

10. When a dermoscopy photo was needed, how often would it be available in the Skin Scan images?
    - ☐ Never
    - ☐ Rarely
    - ☐ Sometimes
    - ☐ Often
    - ☐ Always

11. What cases do you find appropriate for assessment through the Skin Scan Program?
    - ☐ Routine annual skin exam
    - ☐ Follow-up on an existing skin issue
    - ☐ New skin issue
    - ☐ Other (please specify):____

12. Performing skin assessments using the Skin Scan Program was convenient for me in my workflow.
    - ☐ Strongly agree
    - ☐ Agree
    - ☐ Neither agree nor disagree
    - ☐ Disagree
    - ☐ Strongly disagree

13. How likely are you to offer patient participation in the Skin Scan Program again, during the COVID-19 pandemic?
    - ☐ Extremely likely
    - ☐ Likely
    - ☐ Neutral
    - ☐ Unlikely
    - ☐ Extremely unlikely

14. How likely are you to offer patient participation in the Skin Scan Program again, after the COVID-19 pandemic?
    - ☐ Extremely likely
    - ☐ Likely
    - ☐ Neutral
    - ☐ Unlikely
    - ☐ Extremely unlikely

15. How satisfied are you overall with your experience with the Skin Scan Program?
    - ☐ Very satisfied
    - ☐ Satisfied
    - ☐ Neither satisfied nor dissatisfied
    - ☐ Dissatisfied
    - ☐ Very dissatisfied

Please state any additional feedback on your experience with the Skin Scan Program:

____

____

____

____

____
